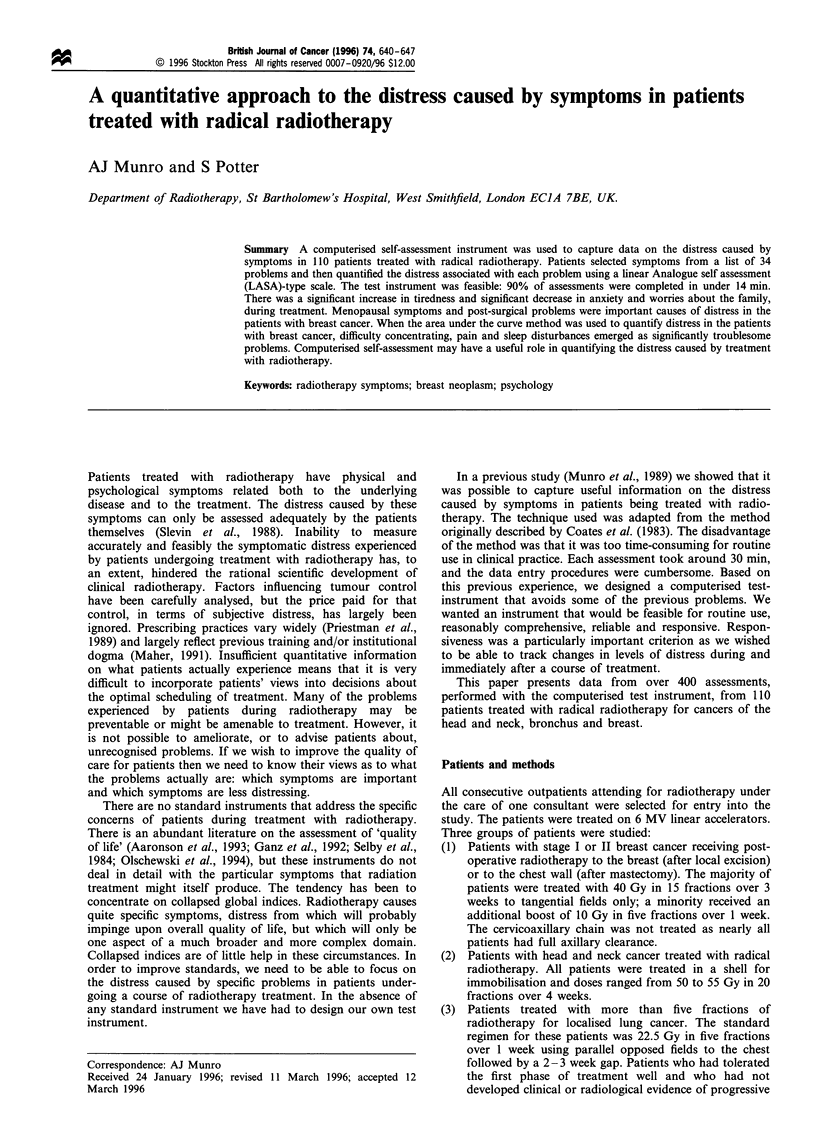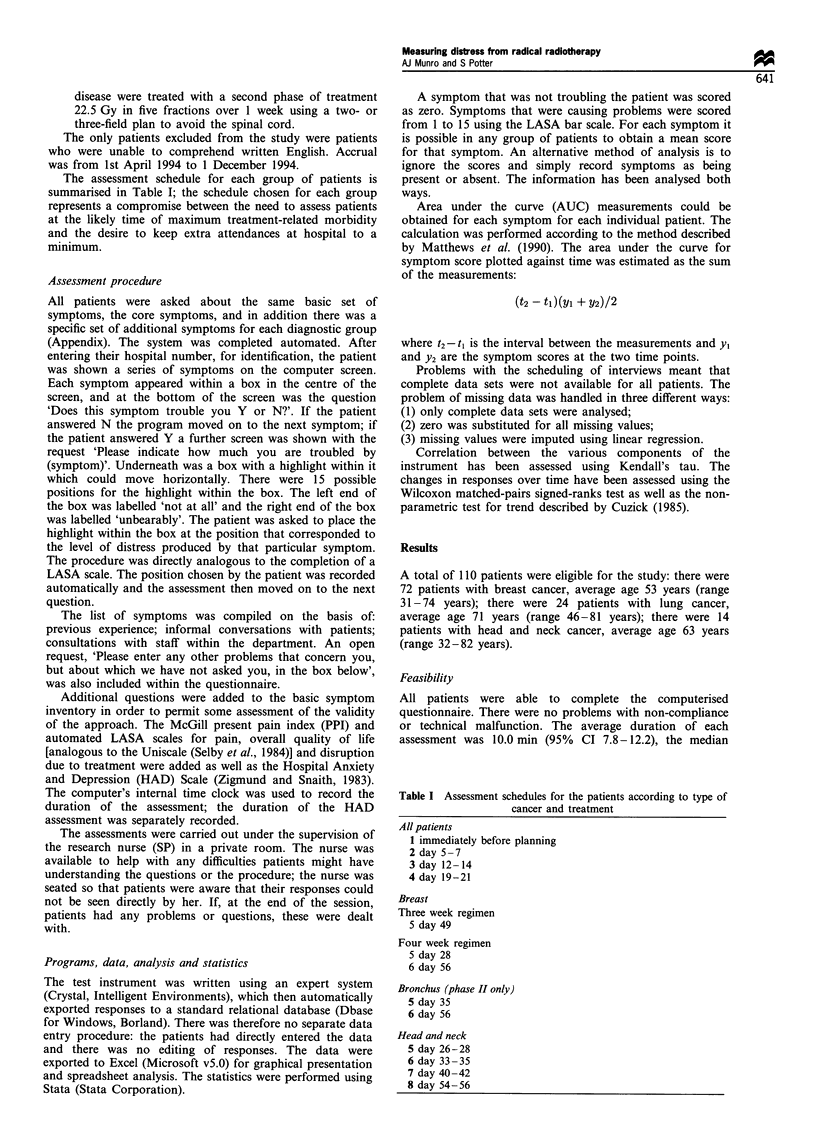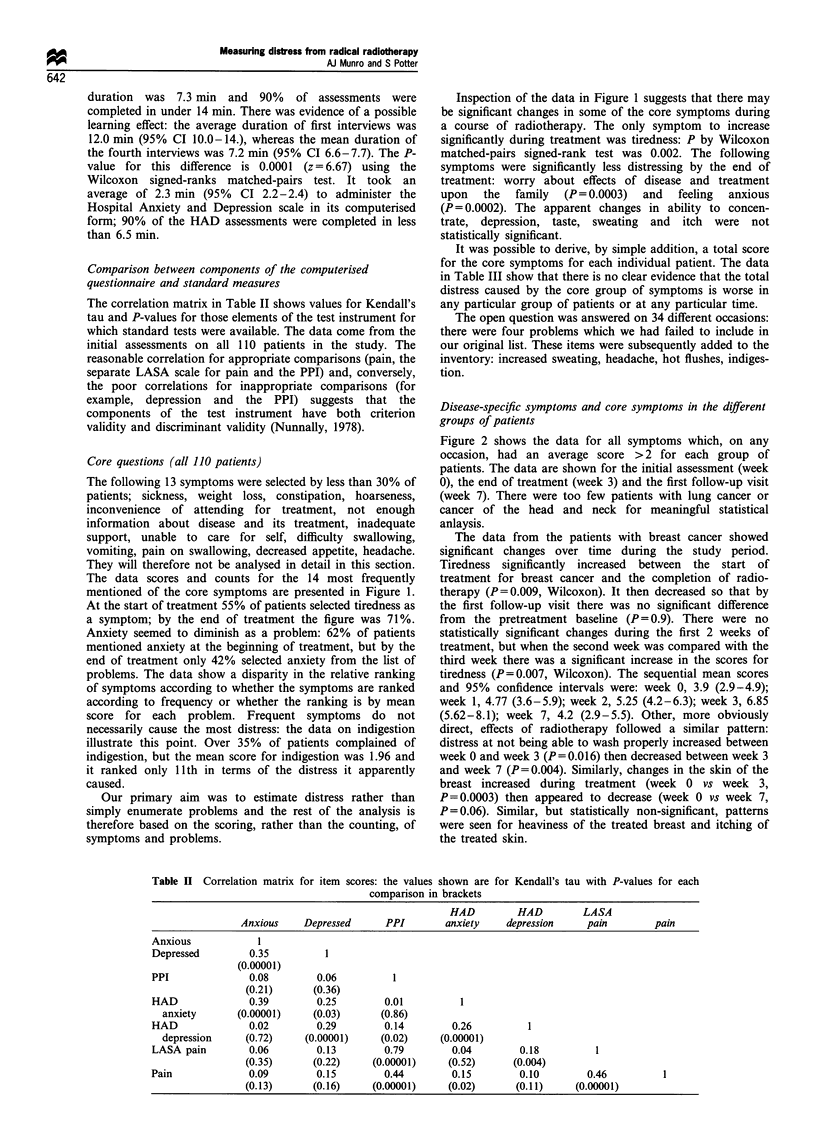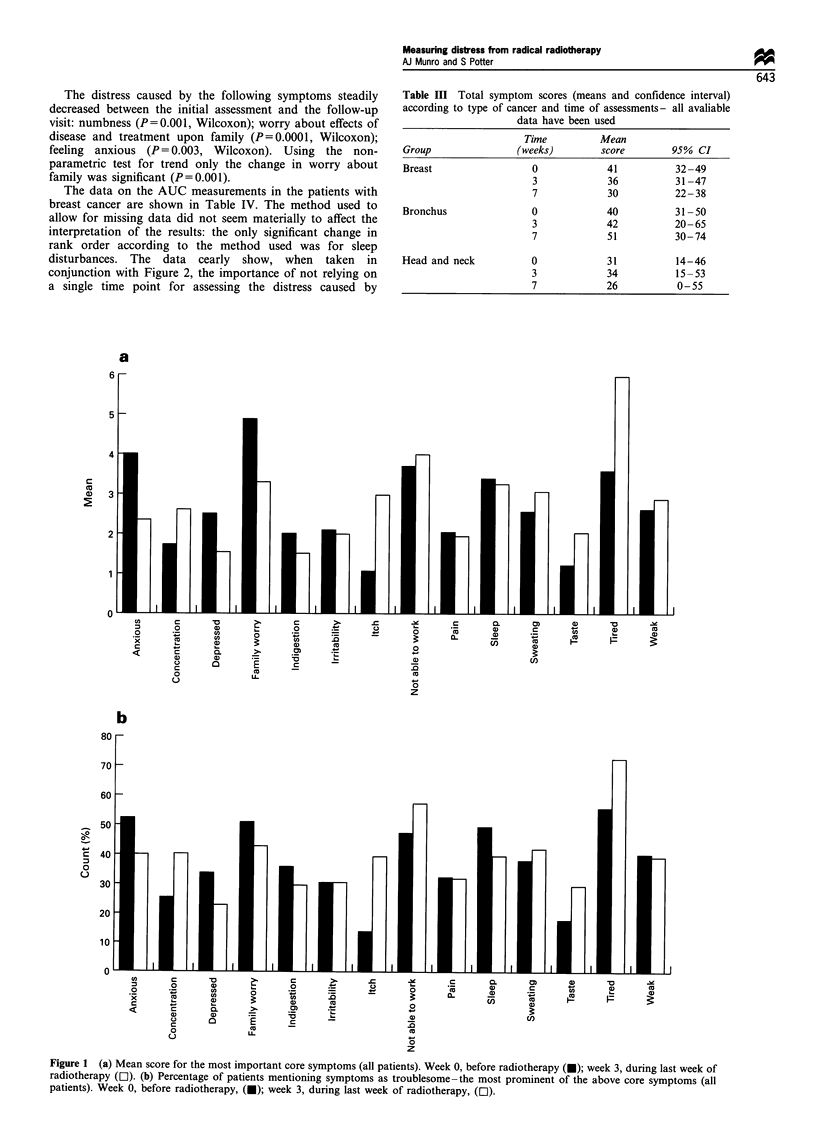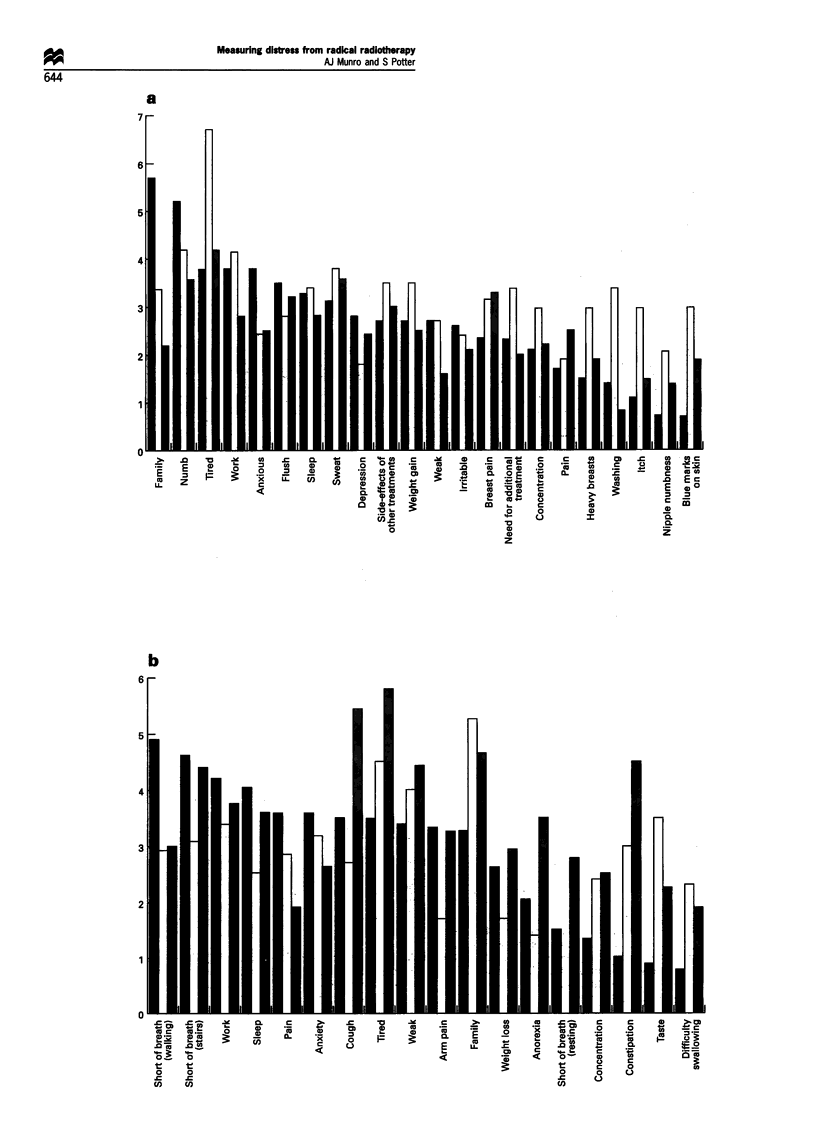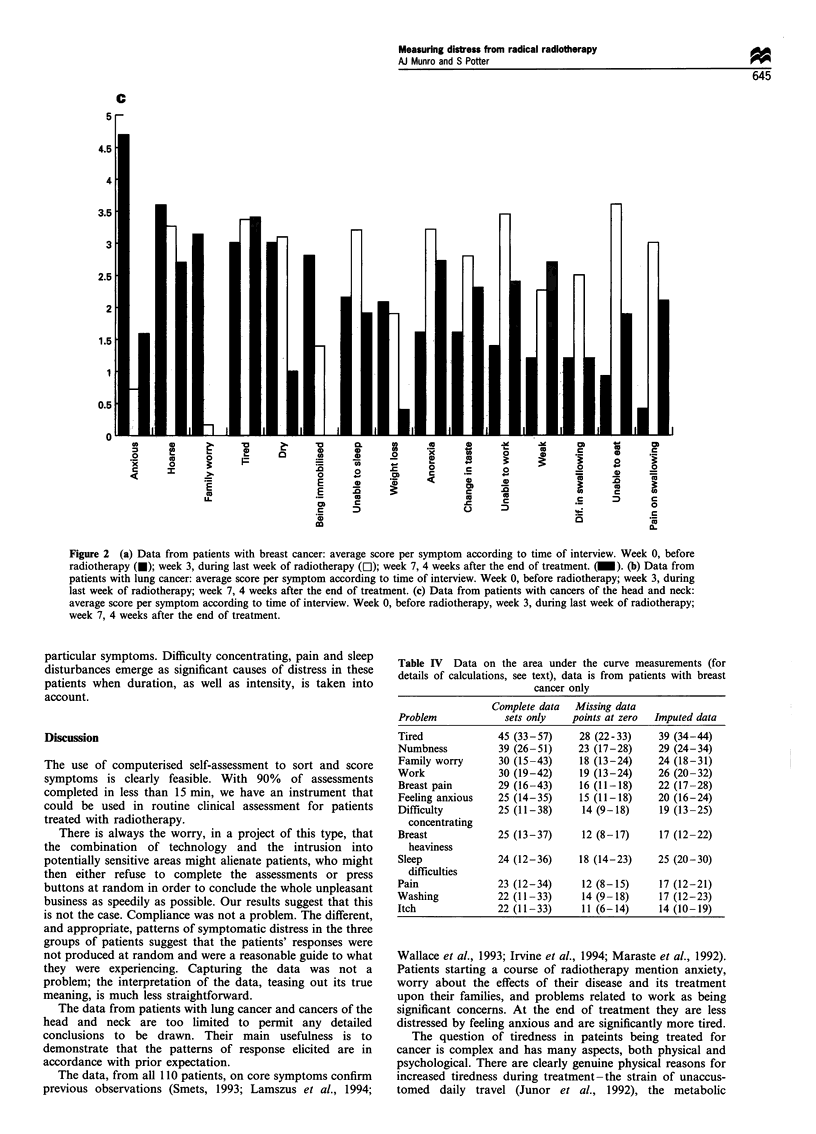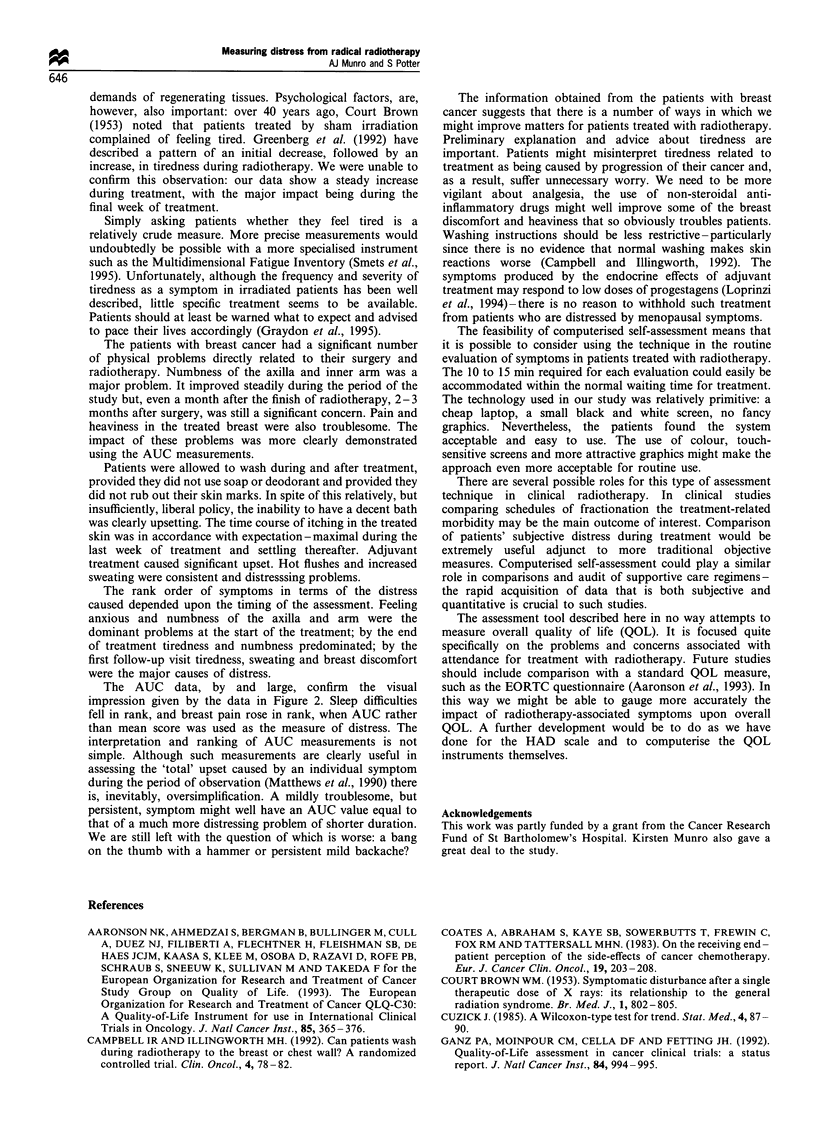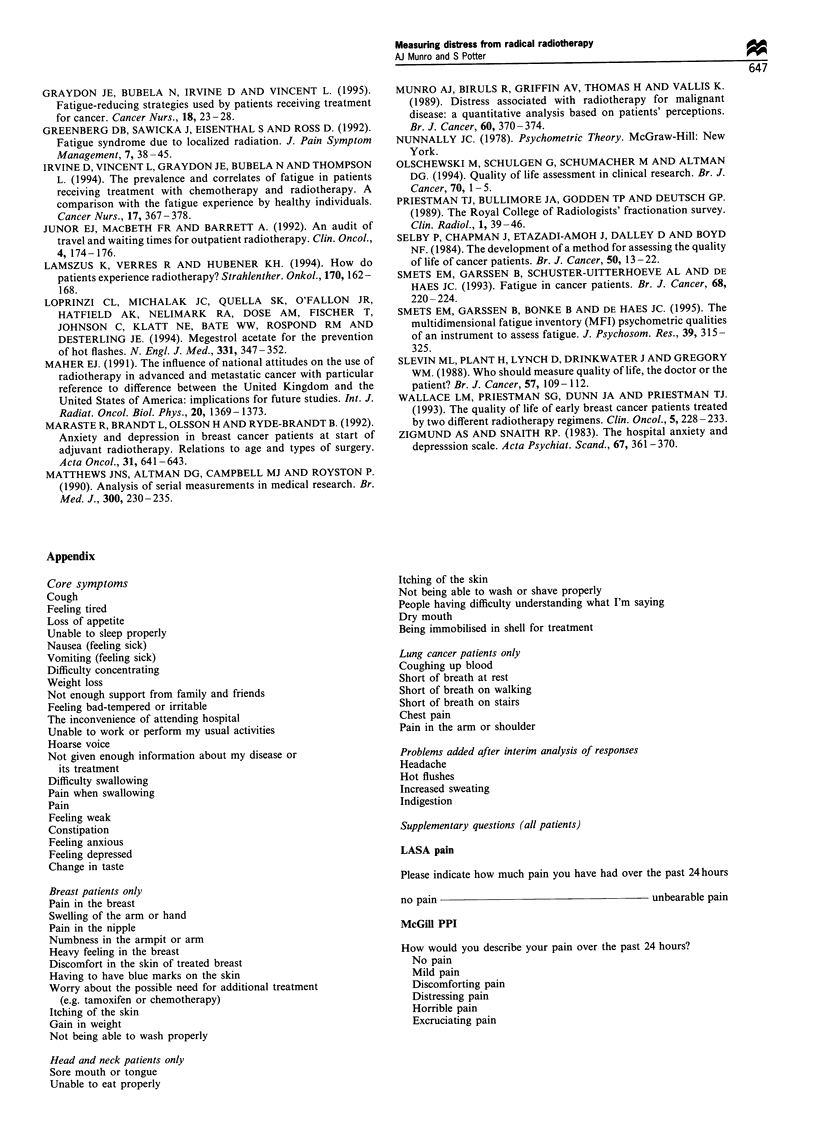# A quantitative approach to the distress caused by symptoms in patients treated with radical radiotherapy.

**DOI:** 10.1038/bjc.1996.414

**Published:** 1996-08

**Authors:** A. J. Munro, S. Potter

**Affiliations:** Department of Radiotherapy, St Bartholomew's Hospital, West Smithfield, London, UK.

## Abstract

A computerised self-assessment instrument was used to capture data on the distress caused by symptoms in 110 patients treated with radical radiotherapy. Patients selected symptoms from a list of 34 problems and then quantified the distress associated with each problem using a linear Analogue self assessment (LASA)-type scale. The test instrument was feasible: 90% of assessments were completed in under 14 min. There was a significant increase in tiredness and significant decrease in anxiety and worries about the family, during treatment. Menopausal symptoms and post-surgical problems were important causes of distress in the patients with breast cancer. When the area under the curve method was used to quantify distress in the patients with breast cancer, difficulty concentrating, pain and sleep disturbances emerged as significantly troublesome problems. Computerised self-assessment may have a useful role in quantifying the distress caused by treatment with radiotherapy.


British Journal of Cancer (1996) 74, 640-647
© 1996 Stockton Press All rights reserved 0007-0920/96 $12.00

### A quantitative approach to the distress caused by symptoms in patients treated with radical radiotherapy

AJ Munro and S Potter

*Department of Radiotherapy, St Bartholomew's Hospital, West Smithfield, London EC1A 7BE, UK.*

**Summary** A computerised self-assessment instrument was used to capture data on the distress caused by symptoms in 110 patients treated with radical radiotherapy. Patients selected symptoms from a list of 34 problems and then quantified the distress associated with each problem using a linear Analogue self assessment (LASA)-type scale. The test instrument was feasible: 90% of assessments were completed in under 14 min. There was a significant increase in tiredness and significant decrease in anxiety and worries about the family, during treatment. Menopausal symptoms and post-surgical problems were important causes of distress in the patients with breast cancer. When the area under the curve method was used to quantify distress in the patients with breast cancer, difficulty concentrating, pain and sleep disturbances emerged as significantly troublesome problems. Computerised self-assessment may have a useful role in quantifying the distress caused by treatment with radiotherapy.

**Keywords:** radiotherapy symptoms; breast neoplasm; psychology

Patients treated with radiotherapy have physical and psychological symptoms related both to the underlying disease and to the treatment. The distress caused by these symptoms can only be assessed adequately by the patients themselves (Slevin *et al.*, 1988). Inability to measure accurately and feasibly the symptomatic distress experienced by patients undergoing treatment with radiotherapy has, to an extent, hindered the rational scientific development of clinical radiotherapy. Factors influencing tumour control have been carefully analysed, but the price paid for that control, in terms of subjective distress, has largely been ignored. Prescribing practices vary widely (Priestman *et al.*, 1989) and largely reflect previous training and/or institutional dogma (Maher, 1991). Insufficient quantitative information on what patients actually experience means that it is very difficult to incorporate patients' views into decisions about the optimal scheduling of treatment. Many of the problems experienced by patients during radiotherapy may be preventable or might be amenable to treatment. However, it is not possible to ameliorate, or to advise patients about, unrecognised problems. If we wish to improve the quality of care for patients then we need to know their views as to what the problems actually are: which symptoms are important and which symptoms are less distressing.

There are no standard instruments that address the specific concerns of patients during treatment with radiotherapy. There is an abundant literature on the assessment of 'quality of life' (Aaronson *et al.*, 1993; Ganz *et al.*, 1992; Selby *et al.*, 1984; Olschewski *et al.*, 1994), but these instruments do not deal in detail with the particular symptoms that radiation treatment might itself produce. The tendency has been to concentrate on collapsed global indices. Radiotherapy causes quite specific symptoms, distress from which will probably impinge upon overall quality of life, but which will only be one aspect of a much broader and more complex domain. Collapsed indices are of little help in these circumstances. In order to improve standards, we need to be able to focus on the distress caused by specific problems in patients undergoing a course of radiotherapy treatment. In the absence of any standard instrument we have had to design our own test instrument.

Correspondence: AJ Munro
Received 24 January 1996; revised 11 March 1996; accepted 12 March 1996

In a previous study (Munro *et al.*, 1989) we showed that it was possible to capture useful information on the distress caused by symptoms in patients being treated with radiotherapy. The technique used was adapted from the method originally described by Coates *et al.* (1983). The disadvantage of the method was that it was too time-consuming for routine use in clinical practice. Each assessment took around 30 min, and the data entry procedures were cumbersome. Based on this previous experience, we designed a computerised test-instrument that avoids some of the previous problems. We wanted an instrument that would be feasible for routine use, reasonably comprehensive, reliable and responsive. Responsiveness was a particularly important criterion as we wished to be able to track changes in levels of distress during and immediately after a course of treatment.

This paper presents data from over 400 assessments, performed with the computerised test instrument, from 110 patients treated with radical radiotherapy for cancers of the head and neck, bronchus and breast.

### Patients and methods

All consecutive outpatients attending for radiotherapy under the care of one consultant were selected for entry into the study. The patients were treated on 6 MV linear accelerators. Three groups of patients were studied:

(1) Patients with stage I or II breast cancer receiving post-operative radiotherapy to the breast (after local excision) or to the chest wall (after mastectomy). The majority of patients were treated with 40 Gy in 15 fractions over 3 weeks to tangential fields only; a minority received an additional boost of 10 Gy in five fractions over 1 week. The cervicoaxillary chain was not treated as nearly all patients had full axillary clearance.
(2) Patients with head and neck cancer treated with radical radiotherapy. All patients were treated in a shell for immobilisation and doses ranged from 50 to 55 Gy in 20 fractions over 4 weeks.
(3) Patients treated with more than five fractions of radiotherapy for localised lung cancer. The standard regimen for these patients was 22.5 Gy in five fractions over 1 week using parallel opposed fields to the chest followed by a 2-3 week gap. Patients who had tolerated the first phase of treatment well and who had not developed clinical or radiological evidence of progressive disease were treated with a second phase of treatment 22.5 Gy in five fractions over 1 week using a two- or three-field plan to avoid the spinal cord.

The only patients excluded from the study were patients who were unable to comprehend written English. Accrual was from 1st April 1994 to 1 December 1994.

The assessment schedule for each group of patients is summarised in Table I; the schedule chosen for each group represents a compromise between the need to assess patients at the likely time of maximum treatment-related morbidity and the desire to keep extra attendances at hospital to a minimum.

*Assessment procedure*

All patients were asked about the same basic set of symptoms, the core symptoms, and in addition there was a specific set of additional symptoms for each diagnostic group (Appendix). The system was completed automated. After entering their hospital number, for identification, the patient was shown a series of symptoms on the computer screen. Each symptom appeared within a box in the centre of the screen, and at the bottom of the screen was the question 'Does this symptom trouble you Y or N?'. If the patient answered N the program moved on to the next symptom; if the patient answered Y a further screen was shown with the request 'Please indicate how much you are troubled by (symptom)'. Underneath was a box with a highlight within it which could move horizontally. There were 15 possible positions for the highlight within the box. The left end of the box was labelled 'not at all' and the right end of the box was labelled 'unbearably'. The patient was asked to place the highlight within the box at the position that corresponded to the level of distress produced by that particular symptom. The procedure was directly analogous to the completion of a LASA scale. The position chosen by the patient was recorded automatically and the assessment then moved on to the next question.

The list of symptoms was compiled on the basis of: previous experience; informal conversations with patients; consultations with staff within the department. An open request, 'Please enter any other problems that concern you, but about which we have not asked you, in the box below', was also included within the questionnaire.

Additional questions were added to the basic symptom inventory in order to permit some assessment of the validity of the approach. The McGill present pain index (PPI) and automated LASA scales for pain, overall quality of life [analogous to the Uniscale (Selby *et al.*, 1984)] and disruption due to treatment were added as well as the Hospital Anxiety and Depression (HAD) Scale (Zigmund and Snaith, 1983). The computer's internal time clock was used to record the duration of the assessment; the duration of the HAD assessment was separately recorded.

The assessments were carried out under the supervision of the research nurse (SP) in a private room. The nurse was available to help with any difficulties patients might have understanding the questions or the procedure; the nurse was seated so that patients were aware that their responses could not be seen directly by her. If, at the end of the session, patients had any problems or questions, these were dealt with.

*Programs, data, analysis and statistics*

The test instrument was written using an expert system (Crystal, Intelligent Environments), which then automatically exported responses to a standard relational database (Dbase for Windows, Borland). There was therefore no separate data entry procedure: the patients had directly entered the data and there was no editing of responses. The data were exported to Excel (Microsoft v5.0) for graphical presentation and spreadsheet analysis. The statistics were performed using Stata (Stata Corporation).

A symptom that was not troubling the patient was scored as zero. Symptoms that were causing problems were scored from 1 to 15 using the LASA bar scale. For each symptom it is possible in any group of patients to obtain a mean score for that symptom. An alternative method of analysis is to ignore the scores and simply record symptoms as being present or absent. The information has been analysed both ways.

Area under the curve (AUC) measurements could be obtained for each symptom for each individual patient. The calculation was performed according to the method described by Matthews *et al.* (1990). The area under the curve for symptom score plotted against time was estimated as the sum of the measurements:

$$(t_2 - t_1)(y_1 + y_2)/2$$

where $t_2 - t_1$ is the interval between the measurements and $y_1$ and $y_2$ are the symptom scores at the two time points.

Problems with the scheduling of interviews meant that complete data sets were not available for all patients. The problem of missing data was handled in three different ways:
(1) only complete data sets were analysed;
(2) zero was substituted for all missing values;
(3) missing values were imputed using linear regression.

Correlation between the various components of the instrument has been assessed using Kendall's tau. The changes in responses over time have been assessed using the Wilcoxon matched-pairs signed-ranks test as well as the non-parametric test for trend described by Cuzick (1985).

### Results

A total of 110 patients were eligible for the study: there were 72 patients with breast cancer, average age 53 years (range 31–74 years); there were 24 patients with lung cancer, average age 71 years (range 46–81 years); there were 14 patients with head and neck cancer, average age 63 years (range 32–82 years).

*Feasibility*

All patients were able to complete the computerised questionnaire. There were no problems with non-compliance or technical malfunction. The average duration of each assessment was 10.0 min (95% CI 7.8–12.2), the median

**Table I** Assessment schedules for the patients according to type of cancer and treatment

| | |
|---|---|
| *All patients* | |
| **1** | immediately before planning |
| **2** | day 5–7 |
| **3** | day 12–14 |
| **4** | day 19–21 |
| *Breast* | |
| Three week regimen | |
| 5 | day 49 |
| Four week regimen | |
| 5 | day 28 |
| 6 | day 56 |
| *Bronchus (phase II only)* | |
| **5** | day 35 |
| **6** | day 56 |
| *Head and neck* | |
| **5** | day 26–28 |
| **6** | day 33–35 |
| **7** | day 40–42 |
| **8** | day 54–56 |

duration was 7.3 min and 90% of assessments were completed in under 14 min. There was evidence of a possible learning effect: the average duration of first interviews was 12.0 min (95% CI 10.0–14.), whereas the mean duration of the fourth interviews was 7.2 min (95% CI 6.6–7.7). The *P*-value for this difference is 0.0001 ($z=6.67$) using the Wilcoxon signed-ranks matched-pairs test. It took an average of 2.3 min (95% CI 2.2–2.4) to administer the Hospital Anxiety and Depression scale in its computerised form; 90% of the HAD assessments were completed in less than 6.5 min.

*Comparison between components of the computerised questionnaire and standard measures*

The correlation matrix in Table II shows values for Kendall's tau and *P*-values for those elements of the test instrument for which standard tests were available. The data come from the initial assessments on all 110 patients in the study. The reasonable correlation for appropriate comparisons (pain, the separate LASA scale for pain and the PPI) and, conversely, the poor correlations for inappropriate comparisons (for example, depression and the PPI) suggests that the components of the test instrument have both criterion validity and discriminant validity (Nunnally, 1978).

*Core questions (all 110 patients)*

The following 13 symptoms were selected by less than 30% of patients; sickness, weight loss, constipation, hoarseness, inconvenience of attending for treatment, not enough information about disease and its treatment, inadequate support, unable to care for self, difficulty swallowing, vomiting, pain on swallowing, decreased appetite, headache. They will therefore not be analysed in detail in this section. The data scores and counts for the 14 most frequently mentioned of the core symptoms are presented in Figure 1. At the start of treatment 55% of patients selected tiredness as a symptom; by the end of treatment the figure was 71%. Anxiety seemed to diminish as a problem: 62% of patients mentioned anxiety at the beginning of treatment, but by the end of treatment only 42% selected anxiety from the list of problems. The data show a disparity in the relative ranking of symptoms according to whether the symptoms are ranked according to frequency or whether the ranking is by mean score for each problem. Frequent symptoms do not necessarily cause the most distress: the data on indigestion illustrate this point. Over 35% of patients complained of indigestion, but the mean score for indigestion was 1.96 and it ranked only 11th in terms of the distress it apparently caused.

Our primary aim was to estimate distress rather than simply enumerate problems and the rest of the analysis is therefore based on the scoring, rather than the counting, of symptoms and problems.

Inspection of the data in Figure 1 suggests that there may be significant changes in some of the core symptoms during a course of radiotherapy. The only symptom to increase significantly during treatment was tiredness: *P* by Wilcoxon matched-pairs signed-rank test was 0.002. The following symptoms were significantly less distressing by the end of treatment: worry about effects of disease and treatment upon the family ($P=0.0003$) and feeling anxious ($P=0.0002$). The apparent changes in ability to concentrate, depression, taste, sweating and itch were not statistically significant.

It was possible to derive, by simple addition, a total score for the core symptoms for each individual patient. The data in Table III show that there is no clear evidence that the total distress caused by the core group of symptoms is worse in any particular group of patients or at any particular time.

The open question was answered on 34 different occasions: there were four problems which we had failed to include in our original list. These items were subsequently added to the inventory: increased sweating, headache, hot flushes, indigestion.

*Disease-specific symptoms and core symptoms in the different groups of patients*

Figure 2 shows the data for all symptoms which, on any occasion, had an average score >2 for each group of patients. The data are shown for the initial assessment (week 0), the end of treatment (week 3) and the first follow-up visit (week 7). There were too few patients with lung cancer or cancer of the head and neck for meaningful statistical anlaysis.

The data from the patients with breast cancer showed significant changes over time during the study period. Tiredness significantly increased between the start of treatment for breast cancer and the completion of radiotherapy ($P=0.009$, Wilcoxon). It then decreased so that by the first follow-up visit there was no significant difference from the pretreatment baseline ($P=0.9$). There were no statistically significant changes during the first 2 weeks of treatment, but when the second week was compared with the third week there was a significant increase in the scores for tiredness ($P=0.007$, Wilcoxon). The sequential mean scores and 95% confidence intervals were: week 0, 3.9 (2.9–4.9); week 1, 4.77 (3.6–5.9); week 2, 5.25 (4.2–6.3); week 3, 6.85 (5.62–8.1); week 7, 4.2 (2.9–5.5). Other, more obviously direct, effects of radiotherapy followed a similar pattern: distress at not being able to wash properly increased between week 0 and week 3 ($P=0.016$) then decreased between week 3 and week 7 ($P=0.004$). Similarly, changes in the skin of the breast increased during treatment (week 0 *vs* week 3, $P=0.0003$) then appeared to decrease (week 0 *vs* week 7, $P=0.06$). Similar, but statistically non-significant, patterns were seen for heaviness of the treated breast and itching of the treated skin.

**Table II** Correlation matrix for item scores: the values shown are for Kendall's tau with *P*-values for each comparison in brackets

| | *Anxious* | *Depressed* | *PPI* | *HAD anxiety* | *HAD depression* | *LASA pain* | *pain* |
|---|---|---|---|---|---|---|---|
| Anxious | 1 | | | | | | |
| Depressed | 0.35 (0.00001) | 1 | | | | | |
| PPI | 0.08 (0.21) | 0.06 (0.36) | 1 | | | | |
| HAD anxiety | 0.39 (0.00001) | 0.25 (0.03) | 0.01 (0.86) | 1 | | | |
| HAD depression | 0.02 (0.72) | 0.29 (0.00001) | 0.14 (0.02) | 0.26 (0.00001) | 1 | | |
| LASA pain | 0.06 (0.35) | 0.13 (0.22) | 0.79 (0.00001) | 0.04 (0.52) | 0.18 (0.004) | 1 | |
| Pain | 0.09 (0.13) | 0.15 (0.16) | 0.44 (0.00001) | 0.15 (0.02) | 0.10 (0.11) | 0.46 (0.00001) | 1 |

The distress caused by the following symptoms steadily decreased between the initial assessment and the follow-up visit: numbness ($P=0.001$, Wilcoxon); worry about effects of disease and treatment upon family ($P=0.0001$, Wilcoxon); feeling anxious ($P=0.003$, Wilcoxon). Using the non-parametric test for trend only the change in worry about family was significant ($P=0.001$).

The data on the AUC measurements in the patients with breast cancer are shown in Table IV. The method used to allow for missing data did not seem materially to affect the interpretation of the results: the only significant change in rank order according to the method used was for sleep disturbances. The data cearly show, when taken in conjunction with Figure 2, the importance of not relying on a single time point for assessing the distress caused by

**Table III** Total symptom scores (means and confidence interval) according to type of cancer and time of assessments– all avaliable data have been used

| *Group* | *Time (weeks)* | *Mean score* | *95% CI* |
|---|---|---|---|
| Breast | 0 | 41 | 32–49 |
| | 3 | 36 | 31–47 |
| | 7 | 30 | 22–38 |
| Bronchus | 0 | 40 | 31–50 |
| | 3 | 42 | 20–65 |
| | 7 | 51 | 30–74 |
| Head and neck | 0 | 31 | 14–46 |
| | 3 | 34 | 15–53 |
| | 7 | 26 | 0–55 |

**Figure 1** (**a**) Mean score for the most important core symptoms (all patients). Week 0, before radiotherapy (■); week 3, during last week of radiotherapy (□). (**b**) Percentage of patients mentioning symptoms as troublesome–the most prominent of the above core symptoms (all patients). Week 0, before radiotherapy, (■); week 3, during last week of radiotherapy, (□).

a

0

1

2

3

4

5

6

7

Family

Numb

Tired

Work

Anxious

Flush

Sleep

Sweat

Depression

Side-effects of other treatments

Weight gain

Weak

Irritable

Breast pain

Need for additional treatment

Concentration

Pain

Heavy breasts

Washing

Itch

Nipple numbness

Blue marks on skin b

0

1

2

3

4

5

6

Short of breath (walking)

Short of breath (stairs)

Work

Sleep

Pain

Anxiety

Cough

Tired

Weak

Arm pain

Family

Weight loss

Anorexia

Short of breath (resting)

Concentration

Constipation

Taste

Difficulty swallowing

**Figure 2** (a) Data from patients with breast cancer: average score per symptom according to time of interview. Week 0, before radiotherapy (■); week 3, during last week of radiotherapy (□); week 7, 4 weeks after the end of treatment. (■). (b) Data from patients with lung cancer: average score per symptom according to time of interview. Week 0, before radiotherapy; week 3, during last week of radiotherapy; week 7, 4 weeks after the end of treatment. (c) Data from patients with cancers of the head and neck: average score per symptom according to time of interview. Week 0, before radiotherapy, week 3, during last week of radiotherapy; week 7, 4 weeks after the end of treatment.

particular symptoms. Difficulty concentrating, pain and sleep disturbances emerge as significant causes of distress in these patients when duration, as well as intensity, is taken into account.

### Discussion

The use of computerised self-assessment to sort and score symptoms is clearly feasible. With 90% of assessments completed in less than 15 min, we have an instrument that could be used in routine clinical assessment for patients treated with radiotherapy.

There is always the worry, in a project of this type, that the combination of technology and the intrusion into potentially sensitive areas might alienate patients, who might then either refuse to complete the assessments or press buttons at random in order to conclude the whole unpleasant business as speedily as possible. Our results suggest that this is not the case. Compliance was not a problem. The different, and appropriate, patterns of symptomatic distress in the three groups of patients suggest that the patients' responses were not produced at random and were a reasonable guide to what they were experiencing. Capturing the data was not a problem; the interpretation of the data, teasing out its true meaning, is much less straightforward.

The data from patients with lung cancer and cancers of the head and neck are too limited to permit any detailed conclusions to be drawn. Their main usefulness is to demonstrate that the patterns of response elicited are in accordance with prior expectation.

The data, from all 110 patients, on core symptoms confirm previous observations (Smets, 1993; Lamszus *et al.*, 1994;

**Table IV** Data on the area under the curve measurements (for details of calculations, see text), data is from patients with breast cancer only

| *Problem* | *Complete data sets only* | *Missing data points at zero* | *Imputed data* |
|---|---|---|---|
| Tired | 45 (33–57) | 28 (22-33) | 39 (34–44) |
| Numbness | 39 (26–51) | 23 (17–28) | 29 (24–34) |
| Family worry | 30 (15–43) | 18 (13–24) | 24 (18–31) |
| Work | 30 (19–42) | 19 (13–24) | 26 (20–32) |
| Breast pain | 29 (16–43) | 16 (11–18) | 22 (17–28) |
| Feeling anxious | 25 (14–35) | 15 (11–18) | 20 (16–24) |
| Difficulty concentrating | 25 (11–38) | 14 (9–18) | 19 (13–25) |
| Breast heaviness | 25 (13–37) | 12 (8–17) | 17 (12–22) |
| Sleep difficulties | 24 (12–36) | 18 (14–23) | 25 (20–30) |
| Pain | 23 (12–34) | 12 (8–15) | 17 (12–21) |
| Washing | 22 (11–33) | 14 (9–18) | 17 (12–23) |
| Itch | 22 (11–33) | 11 (6–14) | 14 (10–19) |

Wallace *et al.*, 1993; Irvine *et al.*, 1994; Maraste *et al.*, 1992). Patients starting a course of radiotherapy mention anxiety, worry about the effects of their disease and its treatment upon their families, and problems related to work as being significant concerns. At the end of treatment they are less distressed by feeling anxious and are significantly more tired.

The question of tiredness in pateints being treated for cancer is complex and has many aspects, both physical and psychological. There are clearly genuine physical reasons for increased tiredness during treatment – the strain of unaccustomed daily travel (Junor *et al.*, 1992), the metabolic demands of regenerating tissues. Psychological factors, are, however, also important: over 40 years ago, Court Brown (1953) noted that patients treated by sham irradiation complained of feeling tired. Greenberg *et al.* (1992) have described a pattern of an initial decrease, followed by an increase, in tiredness during radiotherapy. We were unable to confirm this observation: our data show a steady increase during treatment, with the major impact being during the final week of treatment.

Simply asking patients whether they feel tired is a relatively crude measure. More precise measurements would undoubtedly be possible with a more specialised instrument such as the Multidimensional Fatigue Inventory (Smets *et al.*, 1995). Unfortunately, although the frequency and severity of tiredness as a symptom in irradiated patients has been well described, little specific treatment seems to be available. Patients should at least be warned what to expect and advised to pace their lives accordingly (Graydon *et al.*, 1995).

The patients with breast cancer had a significant number of physical problems directly related to their surgery and radiotherapy. Numbness of the axilla and inner arm was a major problem. It improved steadily during the period of the study but, even a month after the finish of radiotherapy, 2–3 months after surgery, was still a significant concern. Pain and heaviness in the treated breast were also troublesome. The impact of these problems was more clearly demonstrated using the AUC measurements.

Patients were allowed to wash during and after treatment, provided they did not use soap or deodorant and provided they did not rub out their skin marks. In spite of this relatively, but insufficiently, liberal policy, the inability to have a decent bath was clearly upsetting. The time course of itching in the treated skin was in accordance with expectation–maximal during the last week of treatment and settling thereafter. Adjuvant treatment caused significant upset. Hot flushes and increased sweating were consistent and distresssing problems.

The rank order of symptoms in terms of the distress caused depended upon the timing of the assessment. Feeling anxious and numbness of the axilla and arm were the dominant problems at the start of the treatment; by the end of treatment tiredness and numbness predominated; by the first follow-up visit tiredness, sweating and breast discomfort were the major causes of distress.

The AUC data, by and large, confirm the visual impression given by the data in Figure 2. Sleep difficulties fell in rank, and breast pain rose in rank, when AUC rather than mean score was used as the measure of distress. The interpretation and ranking of AUC measurements is not simple. Although such measurements are clearly useful in assessing the 'total' upset caused by an individual symptom during the period of observation (Matthews *et al.*, 1990) there is, inevitably, oversimplification. A mildly troublesome, but persistent, symptom might well have an AUC value equal to that of a much more distressing problem of shorter duration. We are still left with the question of which is worse: a bang on the thumb with a hammer or persistent mild backache?

The information obtained from the patients with breast cancer suggests that there is a number of ways in which we might improve matters for patients treated with radiotherapy. Preliminary explanation and advice about tiredness are important. Patients might misinterpret tiredness related to treatment as being caused by progression of their cancer and, as a result, suffer unnecessary worry. We need to be more vigilant about analgesia, the use of non-steroidal anti-inflammatory drugs might well improve some of the breast discomfort and heaviness that so obviously troubles patients. Washing instructions should be less restrictive–particularly since there is no evidence that normal washing makes skin reactions worse (Campbell and Illingworth, 1992). The symptoms produced by the endocrine effects of adjuvant treatment may respond to low doses of progestagens (Loprinzi *et al.*, 1994)–there is no reason to withhold such treatment from patients who are distressed by menopausal symptoms.

The feasibility of computerised self-assessment means that it is possible to consider using the technique in the routine evaluation of symptoms in patients treated with radiotherapy. The 10 to 15 min required for each evaluation could easily be accommodated within the normal waiting time for treatment. The technology used in our study was relatively primitive: a cheap laptop, a small black and white screen, no fancy graphics. Nevertheless, the patients found the system acceptable and easy to use. The use of colour, touch-sensitive screens and more attractive graphics might make the approach even more acceptable for routine use.

There are several possible roles for this type of assessment technique in clinical radiotherapy. In clinical studies comparing schedules of fractionation the treatment-related morbidity may be the main outcome of interest. Comparison of patients' subjective distress during treatment would be extremely useful adjunct to more traditional objective measures. Computerised self-assessment could play a similar role in comparisons and audit of supportive care regimens–the rapid acquisition of data that is both subjective and quantitative is crucial to such studies.

The assessment tool described here in no way attempts to measure overall quality of life (QOL). It is focused quite specifically on the problems and concerns associated with attendance for treatment with radiotherapy. Future studies should include comparison with a standard QOL measure, such as the EORTC questionnaire (Aaronson *et al.*, 1993). In this way we might be able to gauge more accurately the impact of radiotherapy-associated symptoms upon overall QOL. A further development would be to do as we have done for the HAD scale and to computerise the QOL instruments themselves.

**Acknowledgements**
This work was partly funded by a grant from the Cancer Research Fund of St Bartholomew's Hospital. Kirsten Munro also gave a great deal to the study.

### References

AARONSON NK, AHMEDZAI S, BERGMAN B, BULLINGER M, CULL A, DUEZ NJ, FILIBERTI A, FLECHTNER H, FLEISHMAN SB, DE HAES JCJM, KAASA S, KLEE M, OSOBA D, RAZAVI D, ROFE PB, SCHRAUB S, SNEEUW K, SULLIVAN M AND TAKEDA F for the European Organization for Research and Treatment of Cancer Study Group on Quality of Life. (1993). The European Organization for Research and Treatment of Cancer QLQ-C30: A Quality-of-Life Instrument for use in International Clinical Trials in Oncology. *J. Natl Cancer Inst.*, **85**, 365–376.

CAMPBELL IR AND ILLINGWORTH MH. (1992). Can patients wash during radiotherapy to the breast or chest wall? A randomized controlled trial. *Clin. Oncol.*, **4**, 78–82.

COATES A, ABRAHAM S, KAYE SB, SOWERBUTTS T, FREWIN C, FOX RM AND TATTERSALL MHN. (1983). On the receiving end–patient perception of the side-effects of cancer chemotherapy. *Eur. J. Cancer Clin. Oncol.*, **19**, 203–208.

COURT BROWN WM. (1953). Symptomatic disturbance after a single therapeutic dose of X rays: its relationship to the general radiation syndrome. *Br. Med. J.*, **1**, 802–805.

CUZICK J. (1985). A Wilcoxon-type test for trend. *Stat. Med.*, **4**, 87–90.

GANZ PA, MOINPOUR CM, CELLA DF AND FETTING JH. (1992). Quality-of-Life assessment in cancer clinical trials: a status report. *J. Natl Cancer Inst.*, **84**, 994–995.

GRAYDON JE, BUBELA N, IRVINE D AND VINCENT L. (1995). Fatigue-reducing strategies used by patients receiving treatment for cancer. *Cancer Nurs.*, **18**, 23–28.

GREENBERG DB, SAWICKA J, EISENTHAL S AND ROSS D. (1992). Fatigue syndrome due to localized radiation. *J. Pain Symptom Management*, **7**, 38–45.

IRVINE D, VINCENT L, GRAYDON JE, BUBELA N AND THOMPSON L. (1994). The prevalence and correlates of fatigue in patients receiving treatment with chemotherapy and radiotherapy. A comparison with the fatigue experience by healthy individuals. *Cancer Nurs.*, **17**, 367–378.

JUNOR EJ, MACBETH FR AND BARRETT A. (1992). An audit of travel and waiting times for outpatient radiotherapy. *Clin. Oncol.*, **4**, 174–176.

LAMSZUS K, VERRES R AND HUBENER KH. (1994). How do patients experience radiotherapy? *Strahlenther. Onkol.*, **170**, 162–168.

LOPRINZI CL, MICHALAK JC, QUELLA SK, O'FALLON JR, HATFIELD AK, NELIMARK RA, DOSE AM, FISCHER T, JOHNSON C, KLATT NE, BATE WW, ROSPOND RM AND DESTERLING JE. (1994). Megestrol acetate for the prevention of hot flashes. *N. Engl. J. Med.*, **331**, 347–352.

MAHER EJ. (1991). The influence of national attitudes on the use of radiotherapy in advanced and metastatic cancer with particular reference to difference between the United Kingdom and the United States of America: implications for future studies. *Int. J. Radiat. Oncol. Biol. Phys.*, **20**, 1369–1373.

MARASTE R, BRANDT L, OLSSON H AND RYDE-BRANDT B. (1992). Anxiety and depression in breast cancer patients at start of adjuvant radiotherapy. Relations to age and types of surgery. *Acta Oncol.*, **31**, 641–643.

MATTHEWS JNS, ALTMAN DG, CAMPBELL MJ AND ROYSTON P. (1990). Analysis of serial measurements in medical research. *Br. Med. J.*, **300**, 230–235.

MUNRO AJ, BIRULS R, GRIFFIN AV, THOMAS H AND VALLIS K. (1989). Distress associated with radiotherapy for malignant disease: a quantitative analysis based on patients' perceptions. *Br. J. Cancer*, **60**, 370–374.

NUNNALLY JC. (1978). *Psychometric Theory*. McGraw-Hill: New York.

OLSCHEWSKI M, SCHULGEN G, SCHUMACHER M AND ALTMAN DG. (1994). Quality of life assessment in clinical research. *Br. J. Cancer*, **70**, 1–5.

PRIESTMAN TJ, BULLIMORE JA, GODDEN TP AND DEUTSCH GP. (1989). The Royal College of Radiologists' fractionation survey. *Clin. Radiol.*, **1**, 39–46.

SELBY P, CHAPMAN J, ETAZADI-AMOH J, DALLEY D AND BOYD NF. (1984). The development of a method for assessing the quality of life of cancer patients. *Br. J. Cancer*, **50**, 13–22.

SMETS EM, GARSSEN B, SCHUSTER-UITTERHOEVE AL AND DE HAES JC. (1993). Fatigue in cancer patients. *Br. J. Cancer*, **68**, 220–224.

SMETS EM, GARSSEN B, BONKE B AND DE HAES JC. (1995). The multidimensional fatigue inventory (MFI) psychometric qualities of an instrument to assess fatigue. *J. Psychosom. Res.*, **39**, 315–325.

SLEVIN ML, PLANT H, LYNCH D, DRINKWATER J AND GREGORY WM. (1988). Who should measure quality of life, the doctor or the patient? *Br. J. Cancer*, **57**, 109–112.

WALLACE LM, PRIESTMAN SG, DUNN JA AND PRIESTMAN TJ. (1993). The quality of life of early breast cancer patients treated by two different radiotherapy regimens. *Clin. Oncol.*, **5**, 228–233.

ZIGMUND AS AND SNAITH RP. (1983). The hospital anxiety and depresssion scale. *Acta Psychiat. Scand.*, **67**, 361–370.

### Appendix

*Core symptoms*
Cough
Feeling tired
Loss of appetite
Unable to sleep properly
Nausea (feeling sick)
Vomiting (feeling sick)
Difficulty concentrating
Weight loss
Not enough support from family and friends
Feeling bad-tempered or irritable
The inconvenience of attending hospital
Unable to work or perform my usual activities
Hoarse voice
Not given enough information about my disease or its treatment
Difficulty swallowing
Pain when swallowing
Pain
Feeling weak
Constipation
Feeling anxious
Feeling depressed
Change in taste

*Breast patients only*
Pain in the breast
Swelling of the arm or hand
Pain in the nipple
Numbness in the armpit or arm
Heavy feeling in the breast
Discomfort in the skin of treated breast
Having to have blue marks on the skin
Worry about the possible need for additional treatment (e.g. tamoxifen or chemotherapy)
Itching of the skin
Gain in weight
Not being able to wash properly

*Head and neck patients only*
Sore mouth or tongue
Unable to eat properly
Itching of the skin
Not being able to wash or shave properly
People having difficulty understanding what I'm saying
Dry mouth
Being immobilised in shell for treatment

*Lung cancer patients only*
Coughing up blood
Short of breath at rest
Short of breath on walking
Short of breath on stairs
Chest pain
Pain in the arm or shoulder

*Problems added after interim analysis of responses*
Headache
Hot flushes
Increased sweating
Indigestion

*Supplementary questions (all patients)*

**LASA pain**

Please indicate how much pain you have had over the past 24 hours no pain ———————————— unbearable pain

**McGill PPI**

How would you describe your pain over the past 24 hours?
No pain
Mild pain
Discomforting pain
Distressing pain
Horrible pain
Excruciating pain